# Nickel Wick by Continuous Freeze-Casting: Influences of the Particle Size on the Capillarity and Mechanical Properties

**DOI:** 10.3390/ma14154340

**Published:** 2021-08-03

**Authors:** Pedro Javier Lloreda-Jurado, Laura Chicote, Ernesto Chicardi, Ranier Sepúlveda

**Affiliations:** Departamento de Ingeniería y Ciencia de los Materiales y del Transporte, E.T.S. de Ingeniería, Universidad de Sevilla, Camino de los Descubrimientos, s/n., 41092 Sevilla, Spain; lauchigar@alum.us.es (L.C.); echicardi@us.es (E.C.)

**Keywords:** Ni, wicks, freeze-casting, camphene, polystyrene

## Abstract

The aim of this work was to study the effect of the particle size range, the freeze casting temperature and sintering temperature on the capillarity performance and mechanical properties of Ni wicks manufactured by freeze-casting. The use of Ni/camphene-polystyrene suspensions creates wicks with an open porosity above 80% and average pore sizes of 38 μm to 17 μm by tailoring the particle size ranges and freezing temperatures employed. The incorporation of PS and the use of a continuous freeze-casting process reduces the particle sedimentation and generates a highly interconnected pore structure with regular pore sizes across the sample. The capillarity performances exhibit a fast and complete water adsorption, especially in Ni wicks freeze-casted at 10 °C and sintered at 800 °C, but only when the smaller particle size range is used do Ni wicks achieve sufficient mechanical strength.

## 1. Introduction

The manufacture of porous metal materials has gained significant interest in the scientific community during the last decades due to their low density, good capacity for energy absorption, and high specific surface characteristics. These properties, together with the nature of the material and their pore morphology, give them a great variety of applications, such as battery electrodes [1], sound barriers material [2], filters/catalysts [3,4], biomedical implants [5,6], cushion dampers [7], etc. Furthermore, if the porosity of these materials is interconnected, other attractive characteristics such permeability, infiltration, and heat exchange arise, being the manufacture of wicks one of its most significant applications.

**Figure 1:**
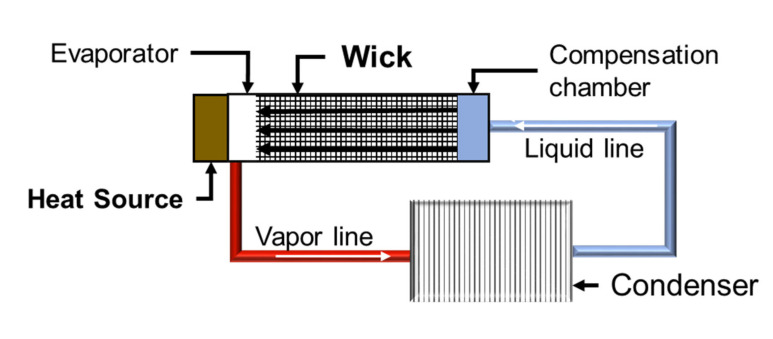
Schema of a loop heat pipe.

Wicks are the main component of loop heat pipe (LHP) systems, highly efficient heat transfer devices quite appreciated in the aerospace and electronics industries [8,9,10]. The wicks allow the coolant to flow self-propelled through its porous structure without the need for an external pump. A schema of the operation of a wick is displayed in Figure 1. LHPs are composed of vapor and liquid lines, condenser, evaporator, compensation chamber, and a wick [11]. Thus, the wick is a porous part with a tailored pore structure, high permeability, improved capillary performance, and made of a low thermal conductivity material such as Ni [12] or stainless steel [13] to reduce the heat transfer. Usually, an open porosity of about 50 to 75% with pore sizes ranges from 1 to 100 µm, and sufficient mechanical strength (above 10 MPa) for dimensional tolerance adjustment is required [9,11].

Traditionally, wicks have been manufactured by cold pressing using space holders [14] or loose sintering technologies [15,16]. As an alternative, the freeze-casting technique deserves special mention due to its numerous advantages, including the possibility of acquiring complex geometries, low cost-effectiveness ratio, eco-friendly, and scalability [8,17]. This technique is based on the direct solidification of a particle suspension, where the solvent crystals push and compact the dispersed particles towards the free spaces of their contour. The resulting porosity of the material is now the space left by the solvent crystals when sublimated. Morphology features and pore size can be altered by: (1) using different solvents—water for lamellar pores or camphene for dendritic crystallization—and (2) the control of thermo-kinetic parameters, such as the solidification rate or thermal gradient [18,19].

In a previous work [17], nickel wicks were manufactured by freeze-casting using NiO nanoparticles of 40 nm in diameter. However, the use of the freeze-casting technique to develop porous metal materials (such as the abovementioned wicks) presents some problems. The high density and the large size (above few microns) of the metallic particles increase the undesirable sedimentation phenomena. Therefore, specific technical variations must be implemented to overcome this disadvantage.

According to a modified version of Stokes’ law [20], the sedimentation rate of a particle in a solution depends on the densities of the particle and the liquid, the dynamic viscosity of the liquid vehicle, and most importantly the particle size. Therefore, this work is focused on the manufacture and characterization of Ni wicks using commercial grade Ni particles implementing these experimental variations: (1) reduction of the metallic particle size through sieving; (2) increasing the liquid camphene viscosity by adding polystyrene of a high molecular weight [21] and, (3) the use of a continuous freeze-casting process to inhibit the particle motion across the sample height. 

## 2. Experiment and Method

### 2.1. Wick Manufacturing

Ni powder (<150 μm in diameter and 99.99% of purity, Sigma Aldrich, St. Louis, MO, USA) was firstly sieved to obtain three different particle size ranges: 44–32 μm, 32–25 μm, and less 25 μm denoted as L (large), M (medium), and S (Small) ranges, respectively. High molecular weight polystyrene (PS, Mw = 350,000 g/mol, Sigma Aldrich, St. Louis, MO, USA) was incorporated into camphene (95% purity, Sigma Aldrich, St. Louis, MO, USA) in a 60 mL seal glass vessel rotating at 100 rpm using a ball milling (lab-made) equipment inside an incubator (INCU-Line IL 23, VWR, Llinars del Vallès, Spain) at 60 °C for approximately 2 h to obtain a camphene-10 vol.% PS solution. Once the PS was completely dissolved, a 10 vol.% of Ni powder with an L, M, or S particle size range was incorporated into the glass vessel and dispersed under the same conditions for 24 h, to achieve Ni/camphene-PS suspensions with those different particle size ranges. A ball-to-powder ratio (BPR) of 1:9 with stainless steel balls of 3 mm in diameter was used during the ball milling process. After dispersion, the suspension was immediately poured into a cylindrical mould made of silicone tube with a copper base precooled inside an incubator (INCU-Line IL 68R, VWR, Llinars del Vallès, Spain) at 10 °C, 25 °C, or 40 °C of freezing temperature ($T_{c}$). The resulting green samples exhibit dimensions of 10 mm in diameter by 26 mm in height, approximately. 

Sublimation of solid camphene within the green samples was completed employing a freeze-dryer (LyoQuest, Telstar, Madrid, Spain) after 24 h at 30 °C under vacuum. Green samples underwent heat treatment at 400 °C for 2 h for organic burn-out followed by a sintering process at 800 °C for 1.5 h in a tubular furnace (Thermolyne 21100, Sigma Aldrich, St. Louis, MO, USA) under reducing conditions with Ar-20H_2_ reducer gas flow (Air Liquide, Seville, Spain) of 0.5 $\mathrm{NL}\cdot \min^{-1}$ to prevent Ni oxidation and enhance the sintering process. An extra batch of samples was also sintered at 600 °C to study the influence of sintering temperature in the porous structure. Heating and cooling rates were set at 1 $℃\cdot \min^{-1}$ and 5 $℃\cdot \min^{-1}$, respectively, to prevent cracks or sample distortions. Final sintered samples measured approximately 8.5 mm in diameter and 22 mm in height.

Sintered samples were denoted as X/YY/ZZZ according to the particle size range (X: L, M, or S), the freezing temperature (YY: 10, 25, or 40 °C), and the sintering temperature (ZZZ: 600 or 800 °C). For example, the L/25/600 sample was fabricated with the L (large particle size range), cooled at 25 °C, and sintered at 600 °C.

### 2.2. Wick Characterization

During freezing, the temperature profile across sample height (h) was recorded using four thermocouples (type T) inserted in the silicone tube at 0, 8, 16, and 24 mm from the mold base. The freezing rate ($FR^{h}$) was obtained as the time to reach freezing temperature $T_{c}$ (40, 25, and 10 °C) at the different h after pouring. Equation (1) shows the calculation procedure:

(1) $$FR^{h}=\frac{60-T_{c}}{t_{h}}\;\left[\frac{℃}{\min}\right]\;,\;\left(h=0,\;8,\;16,\;24\;\mathrm{mm}\right)$$

where $t_{h}$ is the time to reach $T_{c}$ at h when the pouring ends.

The bulk density (ρ), open porosity ($P_{O}$), and close porosity ($P_{C}$) of each sample were determined through Archimedes’ method, which involved soaking the wick samples in hot water for 24 h (tests were repeated 3 times). Several optical microscopy (OM) images were taken using an Eclipse MA100N optical microscope (Nikon, Leuven, Belgium) on the central axial plane at different heights to estimate the average pore size (∅) and wall thickness (τ), and total porosity ($P_{T}$) across the sample height. An area of approximately 5 ${\mathrm{mm}}^{2}$ (with at least 5 OM images) was analyzed at each h to ensure statistical accuracy. OM images were processed using the ImageJ^®^ software (1.53v, U. S. National Institutes of Health, Bethesda, MD, USA) by the non-redundant maximum-sphere-fitted image analysis technique [22].

The capillary performance (CP), i.e., the amount of liquid absorbed over time, was determined for Ni wicks sintered at 800 °C using similar setups reported elsewhere [23,24,25]. The top sample surface was carefully placed in contact with a water reservoir using a micrometric screw. The weight of water adsorbed overtime was ascertained using an electronic analytical balance (Explorer Pro EP114C, OHAUS, Nänikon, Switzerland). Tests were repeated 3 times after drying the samples at 110 °C overnight. The top sample surface was placed in direct contact to simulate the effect of impregnation at the secondary wick and the capillarity suction towards the primary wick. The water mass absorbed fraction was calculated as the mass of water absorbed by the maximum capacity given for the interconnected porosity ($P_{O}$) of the Ni wick.

Uniaxial compression strength tests of the L/25/800, M/25/800, and S/25/800 samples were carried out at room temperature using a universal mechanical testing machine (AG-IS, Shimadzu, Grossenbaum, Germany) with a 10 KN load cell and a crosshead speed of 2 $\mathrm{mm}\cdot \min^{-1}$. Tests were performed on three cylindrical samples of 8 mm in diameter and 4 mm in height which were cut from the sintered samples at the bottom (h = 5.5 mm) and top (h = 16.5 mm) sections. All tests were stopped when a constant plateau on the applied stress is achieved.

## 3. Results and Discussion

### 3.1. Ni Wick Microstructure

According to the temperature profile recorded during continuous freeze-casting, no significant variation of the freezing behavior was observed across the sample height or with the particle size range employed. However, the Ni/camphene-PS suspensions poured at 60 °C did not reach the melting point of camphene at any moment, the suspensions were instantly solidified and the $FR^{h}$ remains constant throughout h. $FR^{h}$ and $t_{h}$ were estimated in 2, 3, and 5 $℃\cdot \min^{-1}$, and 10, 12, and 10 min for the freezing temperature of 40, 25, and 10 °C, respectively. Table 1 shows the bulk density (ρ), open porosity ($P_{O}$), and close porosity ($P_{C}$) of the sintered Ni wicks. Clearly, the ρ was increased with the use of smaller particles and the reduction of $T_{c}$ for samples freeze-casted at 40, 25 and 10 °C. This effect is attributed to the improvement in the particle stacking during freezing with the reduction of the particle size range and the higher thermal gradient applied. The $P_{O}$ was diminished with the reduction of the particle size range, where final $P_{O}$ (72–83%) showed values close to the theoretical maximum of 85%, which correspond to the volume of the fugitive phases (camphene and PS). Therefore, the use of a continuous freeze-casting process prevents most camphene losses. Finally, the $P_{C}$ calculated shows higher values in the samples fabricated with the higher particle size range L. A significant aspect was detailed on sample S/40/800, where the $P_{C}$ was the highest of all sintered samples. This behavior could be attributed to the formation of close pores due to particle coalescence during sintering. The reduction of the sintering temperature to 600 °C produced an overall reduction of the sample ρ and $P_{C}$, with a respective increment in the $P_{O}$.

**Figure 2:**
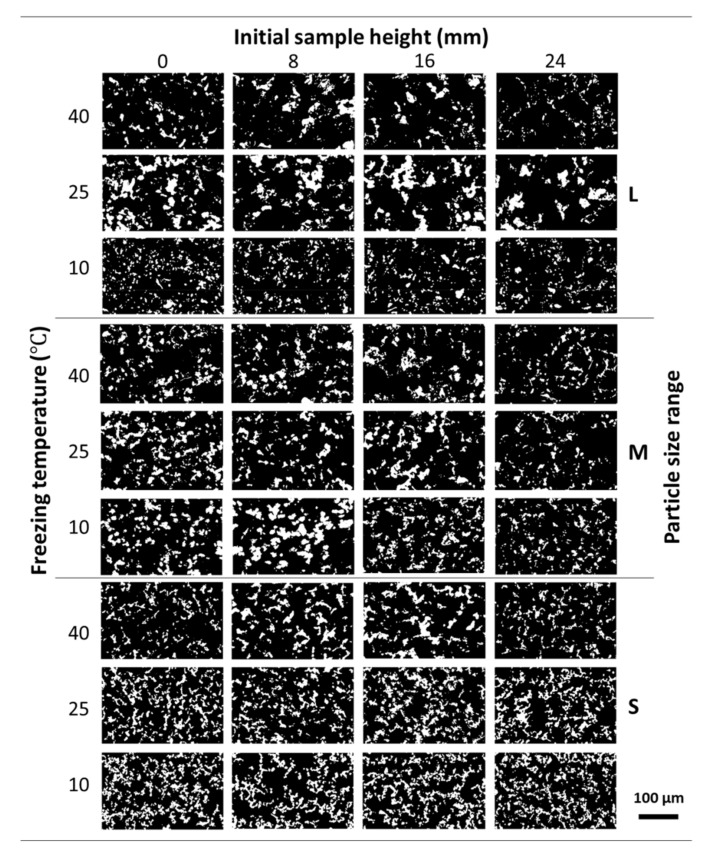
Evolution of the pore structure of the Ni wicks sintered at 800 °C across the sample height. OM micrographs show the effect of the different particle size range (L, M, and S) and freezing temperature (40, 25, and 10 °C) employed. Pores are shown in black and Ni walls were showed in white contrast.

Figure 2 shows the pore structure evolution in the Ni wicks sintered at 800 °C across the sample height when different particle size ranges and freezing temperatures were employed. According to the OM micrographs (black contrast), the pore distribution was highly interconnected, with no significant differences across the sample height regardless of the particle size range or freezing profile employed. The pore size was reduced with the use of the smaller particle size range and especially with the decrease of the freezing temperature. As the particle size diminishes, the critical velocity for particle engulfment increased and the solidification front is enabled to push the particle within the interdendritic spaces [19,26]. Thus, the particles get surrounded by the solid front creating a more continuous pore network with smaller pore sizes. Also, as the number of particles per volume increased due to the particle size reduction and the $T_{c}$ decreased, the branching of the growing solid front is enhanced resulting in much higher interconnectivity of the pores [27].

**Figure 3:**
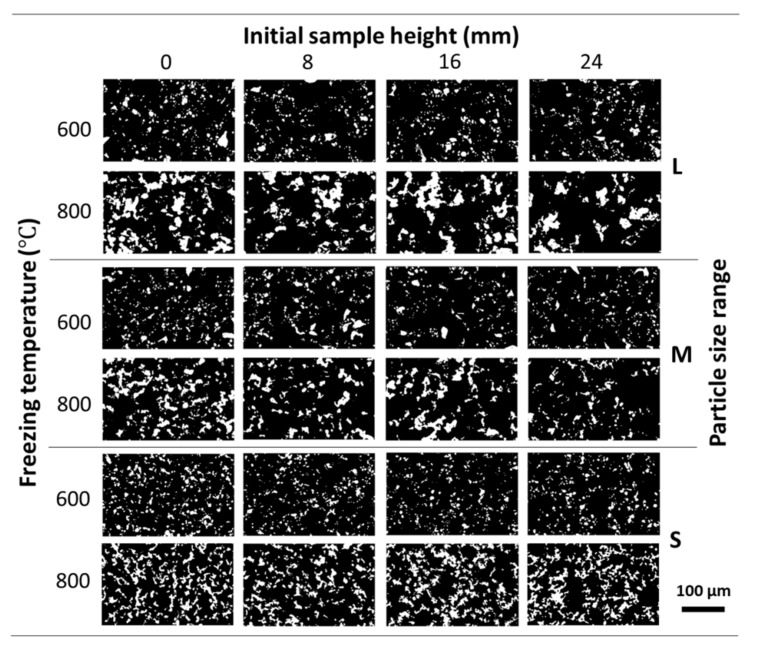
Evolution of the pore structure of the Ni wicks freeze-casted at 25 °C across the initial sample height. OM micrographs show the effect of the different particle size ranges (L, M, and S) and sintering temperatures (600, and 800 °C) employed. Pores are shown in black and Ni walls were in white contrast.

The $P_{T}$ of the Ni Wicks fabricated did not vary significantly across the sample height (Table 2). This behavior could be attributed to the following causes: (1) the lower tendency to sedimentation of the Ni particles due to the increment on the camphene viscosity by the addition of PS [21], which allows the optimal dispersion of Ni particles and, (2) the freezing procedure employed acts as a continuous freeze-casting method, as the maximum temperature recorded by the thermocouple during casting did not go over the camphene melting point, i.e., the Ni/camphene-PS suspensions were solidifying as poured. The samples L, M, S/25/600 show a higher $P_{T}$ as compared with corresponding Ni wick samples sintered at 800 °C due to the inherent lower densification. As $P_{T}$ was calculated using image analysis, no direct correlation could be made with the porosity obtained by Archimedes’ method but can be used to compare the porosity across the sample height. Moreover, the lower sintering temperature diminished the densification process of the Ni particles, as showed Table 1 and Table 2. Figure 3 shows the pore structure evolution of the Ni wick sintered at 600 and 800 °C across the sample height for the different particle sizes range employed. The lower sintering temperature promoted less densification, as the Ni wall (white contrast) looks thinner and scattered.

**Figure 4:**
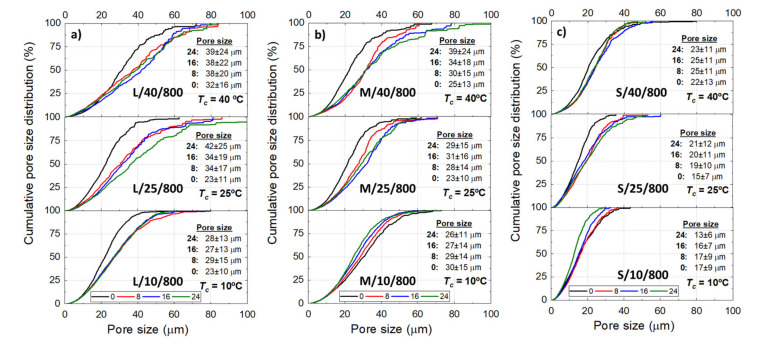
Cumulative pore size distribution of the Ni wick sintered at 800 ℃ according to the initial sample height (h= 0, 8, 16, and 24 mm), freezing temperature ($T_{c}$ = 40, 25, and 10 °C), and de particle size range L (**a**), M (**b**), and S (**c**). Tables inserted show the average pore size (∅) at the respective h.

**Figure 5:**
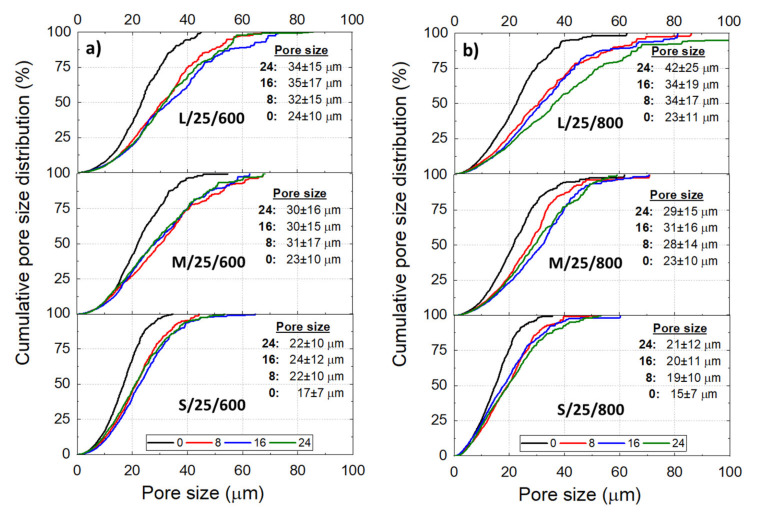
Cumulative pore size distribution of the Ni wicks freeze-casted at 25 °C according to the initial sample height (h = 0, 8, 16, and 24 mm), particle size range (L, M, and S), and the sintering 600 (**a**) and 800 °C (**b**). Tables inserted show the average pore size (∅) at the respective h.

The Ni wicks showed a wide pore size distribution (Figure 4) regardless of the processing parameter. Bottom-parts of Ni wicks L/40-10/800, M/40/800, and M/25/800 presented smaller average pore sizes (∅), and the cumulative distribution moves to lower pore sizes. This behavior corresponds to the zones subjected to a more intense FR where larger particle size ranges promoted the particle engulfment rather than the particle pushing. Overall, as the $T_{c}$ reduces, the cumulative pore size distribution becomes narrow and the average pore size (∅) moves to lower values with the lower particle size range. Samples L/40/800 and S/10/800 showed a ∅ of 38 and 17 μm, respectively and, the 50% cumulative pore size of the Ni wicks exhibited a reduction with $T_{c}$ and the particle size range used. According to Figure 5, Ni wicks exhibited similar ∅ for sintering temperatures of 600 °C (Figure 5a) and 800 °C (Figure 5b). However, wicks sintered at 600 °C did not present sufficient mechanical strength due to the poor particle necking formed during sintering (Figure 3).

### 3.2. Capillary Performance (CP) of the Ni Wicks

**Figure 6:**
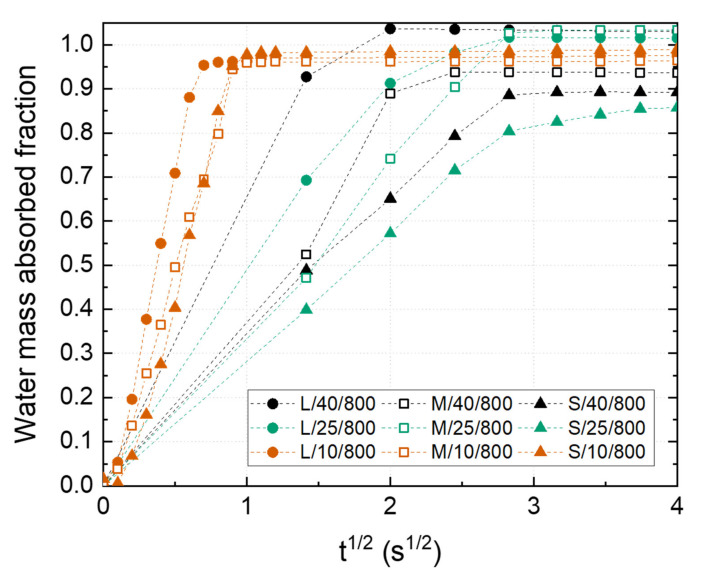
Water mass absorbed fraction of the Ni wicks sintered at 800 °C as a function of the particle size range (S, M, L) and the freezing temperature (10, 25, and 40 °C).

Figure 6 shows the water mass absorbed fraction of the fabricated Ni wicks. A minimum fraction of 0.85 of the total volume was filled during the test in all samples because of the tortuosity of the interconnected porosity (Figure 2), where pore branching and pore section variations impede the liquid transportation. It should be noticed how samples L/40/800, M/40/800, and L/25/800 reach a plate value above 1, which could be attributed to the extra amount of water retained at the wick surfaces, especially those not in contact with the liquid reservoir. This situation was observed in the wicks with bigger pore sizes feeling wet after the capillarity test. Ni wicks freeze-casted at 10 °C have shown a higher mass absorption rate. The presence of small pore size and high pore interconnectivity promoted by the higher FR applied are responsible for this behavior. Similar results were reported elsewhere [17]. Wicks labeled as S/40/800 and S/10/800 showed the lowest water mass absorbed fraction and rate, attributing to the low $P_{O}$ reached leading to minor open porosity since they exhibited similar pore sizes compared with L/10/800 and M/10/800 Ni wick. These results indicated the importance of achieving a high tortuosity pore structure with a small pore size during wick manufacturing to promoted high absorption rates.

### 3.3. Mechanical Properties

**Figure 7:**
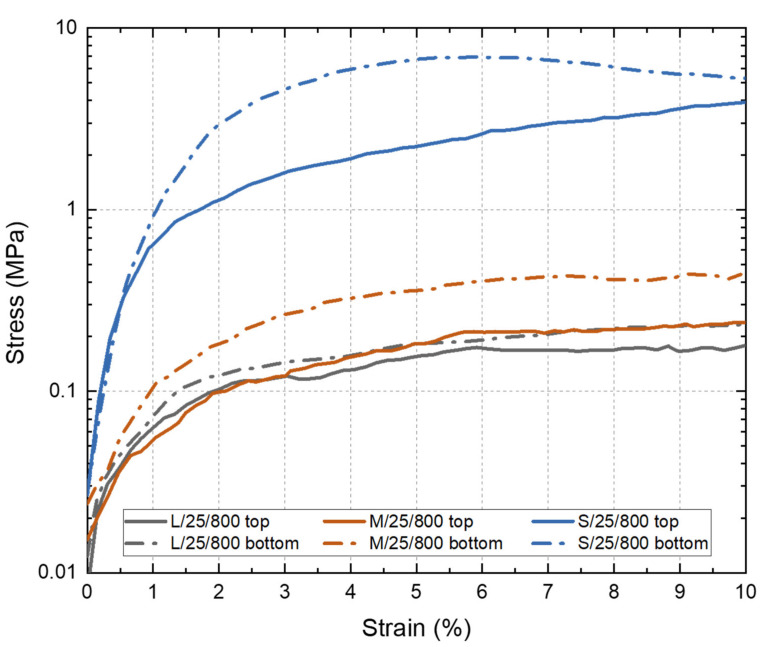
Stress–strain curves of the Ni wicks freeze-casted at 25 °C and sintered at 800 °C according to the particle size range (S, M, and L). Compression tests were performed on samples extracted from the top (full line) and bottom (dotted line) sections of the Ni wicks.

Compression tests were performed in the Ni wicks to evaluate the influence of the $P_{O}$, ∅, and the particle size range employed over the mechanical response. Figure 7 shows the strain-stress curves obtained for the Ni wicks. In general, the use of a smaller particle size range for manufacturing the Ni wicks improved the compression strength. The effects could be attributed to the increment in the bulk density achieved since the particle size range is reduced. In addition, a significant influence on the ∅ can be seen through testing the top and bottom sections of the Ni wicks. All bottom samples have a ∅ smaller than the top samples; therefore, the influence of this pore size reduction is visible as all bottom samples strain-stress curves were above the top-sample curves. L/25/800 and M/25/800 top curves showed similar mechanical behavior due to comparable $P_{O}$ observed for both Ni wicks. In the case of bottom curves of L/25/800 and M/25/800, the particle size reduction increased the mechanical strength, but these Ni-wicks samples showed similar $P_{O}$ and ∅. However, higher densification was obtained in the M/25/800 Ni wick (Table 1), which is responsible for the better mechanical performance. Ni wicks manufactured with the particle size range S and freeze-casted at 25 °C showed the highest compression strength, indeed these samples showed the highest density (2.01 $g\cdot {\mathrm{cm}}^{3}$), and the ∅ was 20 μm and 15 μm at the top and bottom section, respectively. These results indicated that the continuous freezes-casting technique is capable of manufacturing Ni wicks. The ability to use commercial grade Ni particles simplifies the fabrication process of tailored wicks for the electronic industry, where the miniaturization and intricate shapes requirements could be satisfied.

## 4. Conclusions

The addition of PS as a camphene thickening additive and the use of a continuous freeze-casting method has proved successful to manufacture functional Ni wicks with a minimum of 80% of open porosity and almost uniform pore sizes. Pore size and pore morphology was influenced by the particles size used and the FR applied during the freeze-casting process. The pore morphology and the pore tortuosity seem to be the main factors to tailor the Ni-wick capillary performance. The use of higher FR developed Ni-wicks with a faster absorption rate, regardless of the particle size employed. Therefore, Ni wicks L/10/800, M/10/800, and S/10/800 showed a better capillarity performance with the use of commercial grade Ni particle however, only the Ni wick S/10/800 complies with the mechanical property requirements.

## Figures and Tables

**Table 1:** Bulk density (ρ), open ($P_{O}$), and close ($P_{C}$) porosity of the sintered Ni wicks.

Sample	ρ $\;(g\cdot cm^{-3})$	$P_{O}\;(\%)$	$P_{C}\;(\%)$
L/40/800	1.09	81	7
L/25/800	1.22	81	5
L/10/800	1.48	78	5
M/40/800	1.24	83	3
M/25/800	1.29	81	4
M/10/800	1.51	79	4
S/40/800	1.65	72	9
S/25/800	2.01	75	2
S/10/800	2.09	75	1
L/25/600	1.11	83	4
M/25/600	1.16	84	3
S/25/600	1.38	83	1

**Table 2:** Total porosity ($P_{T}$) of the sintered Ni wicks across the initial sample height.

Sample	$P_{T}$ (%) According to the Initial Sample Height (mm)			
	0	8	16	24
L/40/800	86	85	85	86
L/25/800	83	84	84	85
L/10/800	87	89	89	88
M/40/800	89	89	89	93
M/25/800	85	86	87	89
M/10/800	83	85	86	88
S/40/800	86	84	82	86
S/25/800	79	80	80	81
S/10/800	77	77	77	76
L/25/600	90	94	93	93
M/25/600	92	92	93	93
S/25/600	87	91	93	92

## Data Availability

The data presented in this study are available on request from the corresponding author.
